# Leg versus Arm Vaccination

**Published:** 1896-03-07

**Authors:** Norman Porritt

**Affiliations:** Hon. Surgeon Huddersfield Infirmary.


### Medical Progress and Hospital Clinics.

[*The Editor will be glad to receive offers of co-operation and contributions from members of the profession. All letters should be addressed to* The Editor, at the Office, 428, Strand, London, W.C.]

### LEG *VERSUS* ARM VACCINATION.

By Norman Porritt, M.R.C.S., L.R.C.P., Hon. Surgeon Huddersfield Infirmary.

Customs once established are difficult to alter, and the almost universal practice of vaccinating on the arm seems to be a sufficient reason for adopting no other. But the writer ventures to affirm that the leg is a better part upon which to place the vesicles than the arm.

In the first place, there is the æsthetic reason, which applies particularly to girls, who are spared the necessity of appearing with scarred shoulders whenever fashion ordains the wearing of low dresses. To some this consideration is of some importance. Then the little operation is done more easily upon the leg. Placing the highest puncture on the outer aspect of the leg, midway between the knee and the ankle, the foot and ankle can be grasped and the limb steadied. In arm vaccination the shoulder moves with every movement and wriggle of the child's trunk. Hence, in the case of the leg, there is less pain, the child is quieter, and the operation is sooner over. This is especially the case if the vaccination be done when the child is about six weeks old—at an age when it spends much time in sleep. The napkin clears the highest vesicle, and in practice—though in theory probable—the urine never gets near to irritate the vesicles.

If care be taken not to place the vesicles too near the posterior surface of the limb the vigorous movements of a healthy baby are not interfered with, and a child with fully developed vesicles on the outside of its leg may be seen crowing and throwing its legs about as only a happy baby can.

For, one advantage of leg vaccination is that there is less inflammatory reaction, due to the fact that in arm vaccination the dressing and undressing of the child—necessitating the removal of the sleeves of the night-dress or frock—no matter how carefully done irritate the vesicles. In leg vaccination there is nothing to pull over or across the vesicles, for the top of the child's little wool boot scarcely touches them.

Should, however, excessive reaction come on it is an advantage to have it at some distance from the trunk. When a vaccination case "goes wrong," with spread of inflammation and infiltration of the parts around, the inflammatory action soon spreads from the arm to the trunk, where it is far more dangerous to a young baby than if it were confined to a limb.

Upon whatever part of the body vaccination is done its protective influence is the same, and mothers, who have had experience of leg as well as arm vaccination, prefer the former as giving less trouble, both to themselves and the baby.

If it is urged that leg vaccination may cause some medical examiner to put down cases as unvaccinated because there are no marks on the arm, it is only necessary for leg vaccination to be more generally adopted and better known, to make men, in examining cases, look at the leg as well as the arm; and if the advantages were better known that time would not be long in coming.

